# Loss of prostasin (PRSS8) in human bladder transitional cell carcinoma cell lines is associated with epithelial-mesenchymal transition (EMT)

**DOI:** 10.1186/1471-2407-9-377

**Published:** 2009-10-22

**Authors:** Li-Mei Chen, Nicole J Verity, Karl X Chai

**Affiliations:** 1 Department of Molecular Biology and Microbiology, Burnett School of Biomedical Sciences, University of Central Florida College of Medicine, Orlando, Florida 32816, USA; 2 Biomolecular Science Center, Burnett School of Biomedical Sciences, University of Central Florida College of Medicine, Orlando, Florida 32816, USA

## Abstract

**Background:**

The glycosylphosphatidylinositol (GPI)-anchored epithelial extracellular membrane serine protease prostasin (PRSS8) is expressed abundantly in normal epithelia and essential for terminal epithelial differentiation, but down-regulated in human prostate, breast, and gastric cancers and invasive cancer cell lines. Prostasin is involved in the extracellular proteolytic modulation of the epidermal growth factor receptor (EGFR) and is an invasion suppressor. The aim of this study was to evaluate prostasin expression states in the transitional cell carcinomas (TCC) of the human bladder and in human TCC cell lines.

**Methods:**

Normal human bladder tissues and TCC on a bladder cancer tissue microarray (TMA) were evaluated for prostasin expression by means of immunohistochemistry. A panel of 16 urothelial and TCC cell lines were evaluated for prostasin and E-cadherin expression by western blot and quantitative PCR, and for prostasin gene promoter region CpG methylation by methylation-specific PCR (MSP).

**Results:**

Prostasin is expressed in the normal human urothelium and in a normal human urothelial cell line, but is significantly down-regulated in high-grade TCC and lost in 9 (of 15) TCC cell lines. Loss of prostasin expression in the TCC cell lines correlated with loss of or reduced E-cadherin expression, loss of epithelial morphology, and promoter DNA hypermethylation. Prostasin expression could be reactivated by demethylation or inhibition of histone deacetylase. Re-expression of prostasin or a serine protease-inactive variant resulted in transcriptional up-regulation of E-cadherin.

**Conclusion:**

Loss of prostasin expression in bladder transitional cell carcinomas is associated with epithelial-mesenchymal transition (EMT), and may have functional implications in tumor invasion and resistance to chemotherapy.

## Background

According to the American Cancer Society Cancer Facts & Figures 2008, 68,810 new cases of bladder cancer would have been diagnosed in the United States over the year of 2008, with a total of 14,100 bladder cancer patients dying from the disease. The cost of managing bladder cancer and the associated complications is estimated to be $65,158 per patient per year in the US [1], amounting to a multi-billion dollar economic impact. For bladder cancer patients of all stages, the 5-year survival rate is 80%. For localized disease, the 5-year survival rate is 92%. But the 5-year survival rate sharply declines to 45% and 6% for patients with regional and distant metastasis, respectively. The majority of bladder cancers (90%) are "transitional cell carcinomas" (TCC) [2]. More than 70-80% of the bladder cancers are papillary non-invasive tumors that rarely develop into invasive tumors. The remaining bladder cancer cases (20-30%) are non-papillary invasive tumors that produce lymphatic and distant metastasis, accounting for most of the bladder cancer deaths. Radical cystectomy combined with chemo- or radiation therapy is required for patients with invasive bladder cancers, and offers improved survival [3]. But 50% of patients with invasive bladder tumors die from metastasis within 2 years of diagnosis [4,5]. For patients with metastasis, i.e., invasive tumors that escaped the current chemo- or radiation adjuvant therapy, new drugs and new drug targets are needed.

Recent advances in bladder cancer research have identified the process of epithelial-mesenchymal transition (EMT) as an important factor in determining patient responses to therapy and survival. EMT is causal to the development of invasive and metastatic cancers including TCC. At the molecular level, the epidermal growth factor receptor (EGFR), the cell adhesion molecule E-cadherin, and transcription repressors of E-cadherin such as SNAIL and SLUG have been shown to play essential and major roles in EMT and development of invasive and metastatic bladder cancer [6]. We have shown that a glycosylphosphatidylinositol (GPI)-anchored epithelial extracellular membrane serine protease, prostasin/PRSS8, modulates EGFR signalling via enhancement of matriptase cleavage of the EGFR extracellular domain (ECD), and regulates SLUG and E-cadherin expression in cancer cells [7,8]. Prostasin is essential for terminal epithelial differentiation [9] and is abundantly expressed in the normal epithelium [10]. In epithelial cancers, prostasin expression, however, is down-regulated. Down-regulation of prostasin protein expression has been shown for high-grade and hormone-refractory prostate cancers [11,12], breast cancers [13], and gastric cancers [14]. Promoter DNA hypermethylation was shown to be a mechanism of prostasin silencing in various cancer cell lines [14-16]. Invasive human cancer cell lines are often associated with loss of prostasin expression while prostasin re-expression inhibits their invasion through the Matrigel [11,15]. In ovarian cancers, however, an up-regulation of prostasin was reported [17].

We have previously demonstrated urothelial-specific prostasin expression in the mouse bladder [18]. But the expression states of prostasin in urothelial cancers have not been evaluated to date. In this study, we undertook this task with an immunohistochemical examination of prostasin protein expression in transitional cell carcinomas (TCC) using a commercial bladder cancer tissue microarray (TMA). We further evaluated prostasin expression in a normal human urothelial cell line (UROtsa) and 15 TCC cell lines. The methylation states at the -96 CpG dinucleotide in the prostasin gene promoter region were determined in all the cell lines.

## Methods

### Immunohistochemical (IHC) evaluation of prostasin expression in bladder cancer tissue microarray (TMA)

A paraffin slide panel containing normal human tissues including the bladder (Multi-Tissue VI) was obtained from BioChain Institute, Inc. (Hayward, CA). A Bladder Carcinoma TMA (ARY-HH0087) containing 80 tissue cores, each at 1.5 mm in diameter; with 40 cancer tissues and 40 matching or independent non-cancerous tissues was obtained from Folio Biosciences (Columbus, OH). The tissue procurement by these commercial suppliers was conducted with informed consent for use in research, in compliance with the Helsinki Declaration; also with approval of the Institutional Review Board (IRB) at the institutions where the tissues were collected. The tissues on the slides from these commercial sources did not contain any personal identifiers. The use of these tissues in this study is exempt from reviews by the IRB of the authors' institution, per Code of Federal Regulations Title 45, Part 46, Section 101 (United States Department of Health and Human Services). The IHC staining with a prostasin-specific polyclonal antibody was carried out as described previously [11]. The IHC images were taken with various objective lenses and a SONY DXC-950 3CCD camera using a 0.45× coupler on a Zeiss Axioskop 2 microscope.

Prostasin-positive staining in the TMA tissue cores was assigned a score of "1", defined as contiguous prostasin-specific staining in the urothelial cells. Prostasin-negative staining in the TMA tissue cores was assigned a score of "0", including tissues presenting sporadic prostasin-positive cells in areas of overall negative staining. These scores were used to calculate the average prostasin staining score/percent positive in each tumor grade group and the non-cancerous group. The IHC scores were evaluated by single-factor ANOVA (analysis of variance) with ranked prostasin staining data (rank 1 = no staining, rank 2 = positive staining), using the ANOVA data analysis tool of Microsoft Excel 2003. The Tukey-Kramer method was used for post hoc analysis of the ANOVA data to evaluate between-group differences.

### Cell cultures

The normal immortalized urothelial cell line UROtsa was kindly provided by Dr. Donald A. Sens of the University of North Dakota School of Medicine, Grand Forks, ND, and cultured as described previously [19]. The TCC cell lines HT-1376, J82, RT4, T24, UM-UC-3 were from the American Type Culture Collection (Manassas, VA) and cultured in RPMI-1640 medium supplemented with 10% fetal bovine serum (FBS). The KU-7 cell line was kindly provided by Dr. Charles J. Rosser of the University of Florida School of Medicine, Gainesville, FL, and cultured in RPMI-1640/10% FBS. A panel of TCC cell lines from the MD Anderson Cancer Center (Houston, TX), including 253J P and 253J B-V (kindly provided by Dr. Colin P. N. Dinney), UM-UC-5, -6, -9, -10, -12, -13, and -14 (kindly provided by Dr. H. Barton Grossman), were cultured in EMEM supplemented with 10% FBS, 1× glutamine, 1× vitamins, 1× non-essential amino acids, and 1× sodium pyruvate (all from the Invitrogen Corporation, Carlsbad, CA). Sub-confluent cultures of the urothelial cells were used for phase-contrast photography with a SONY DXC-950 3CCD camera using a 0.45× coupler on a Zeiss Axioskop 2 microscope, under a 10× objective lens.

### Western blot analysis

The procedures for western blot analysis were as described previously [20]. Briefly, cells were washed with 1× PBS and lysed at 4°C for 15 minutes in RIPA buffer. The supernatant was collected following centrifugation at 10,000 × g for 10 minutes. Protein concentrations were determined using a DC Protein Assay (Bio-Rad, Hercules, CA). Equal amounts of total protein for each sample were resolved on SDS-PAGE and electro-transferred to a nitrocellulose membrane. The membranes were blocked with 5% non-fat milk in TBS-T (20 mM Tris-HCl, pH 7.4, 150 mM NaCl, 0.1% Tween-20), and incubated with appropriate primary antibodies. The primary antibodies used were human prostasin ([20], used at 1:4,000), E-cadherin (BD Biosciences, San Jose, CA; used at 1:3,000), and glyceraldehyde-3-phosphate dehydrogenase (GAPDH, Santa Cruz Biotechnology, Inc., Santa Cruz, CA; used at 1:5,000). After incubation with each primary antibody, the membranes were washed and incubated for 1 hour with an appropriate secondary antibody conjugated to the horseradish peroxidase (HRP) (Promega, Madison, WI; 1:10,000). The membranes were then washed and subjected to enhanced-chemiluminescence reaction (ECL, Pierce Biotechnology, Inc., Rockford, IL) before exposure to X-ray films.

### Analysis of prostasin promoter CpG methylation state by methylation-specific PCR (MSP)

High molecular weight genomic DNA was extracted from the urothelial cells as described previously [15]. For each cell line, aliquots of 2 μg of DNA were separately digested with 5 units of Xho I (**X**), Hpa II (**H**), or Msp I (**M**) (Invitrogen) in a total volume of 20 μl at 37°C for overnight. The digested DNA was diluted to 100 μl with water (at a final concentration of 20 ng/μl). MSP was carried out with 100 ng of the digested DNA using the following primers specific to the prostasin promoter region: upstream - 5'-CAC ATA CAC ACT ACA CAC CG-3'; and down-stream - 5'-TGG CTG CAC CTA CCT GCC CG-3'. The upstream primer ends with part of the Hpa II/Msp I sequence at the -96 CpG: CCGG (underlined). The reaction mixtures were placed in thin-walled 0.5-ml microcentrifuge tubes, overlaid with mineral oil, and subjected to the following thermal cycling program: 94°C/90 seconds → 28× [94°C/30 seconds → 60°C/45 seconds → 72°C/45 seconds]. The PCR products were resolved in 1% agarose gels and stained with ethidium bromide, and photographed. The images were black-white inverted for greater contrast. The amplicon was verified by DNA sequencing.

### Reactivation of prostasin expression in the TCC cells by demethylation and histone deacetylase inhibition

KU-7 cells were cultured in 12-well plates at a density of 2.5 × 10^5^/well for overnight, and then treated with 500 nM 5-aza-2'-deoxycytidine (**5-Aza-2'-dC**) for 24 hours. Trichostatin A (**TSA**) at 1 μM or equal volumes of 95% ethanol (**E**) (solvent control for TSA) was then added to the cells treated with 5-Aca-2'-dC, or control cells, which were incubated for another 24 hours before western blot analysis.

### Re-expression of prostasin and a serine active-site mutant variant in KU-7

The ViraPower™ Lentiviral Expression System (Invitrogen) was used for generating replication-incompetent lentivirus stably expressing a recombinant wild-type human prostasin (Lenti4-Pro) or a serine active-site mutant (Lenti4-ProM) using the appropriate cDNA's described previously [8]. KU-7 cells were seeded at 30% confluence in a T-25 flask in preparation for lentiviral infection. On the next day, the Lenti4-LacZ (Invitrogen), Lenti4-Pro, or Lenti4-ProM virus was added for infection in the culture medium according to Invitrogen protocols. The transfected colonies were selected with zeocin (at a final concentration of 1 μg/ml). Approximately 200 colonies were pooled for each polyclonal subline, KU-7/LacZ, KU-7/Pro, and KU-7/ProM.

### Reverse-transcription and real-time quantitative polymerase chain reaction (qRT-PCR)

The procedures for qRT-PCR analysis of prostasin, E-cadherin, EGFR, and GAPDH mRNA expression have been described previously [8,18].

## Results

### The epithelial glycosylphosphatidylinositol (GPI)-anchored serine protease, prostasin, is expressed in the normal urothelium but down-regulated in high-grade transitional cell carcinomas

**Figure 1:**
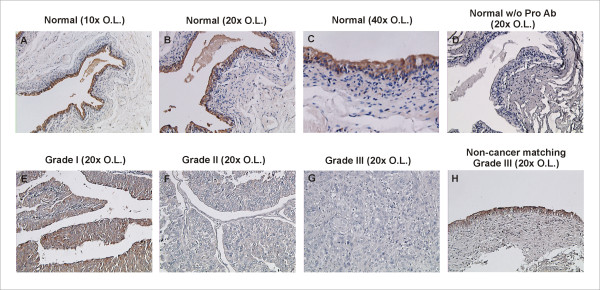
**Prostasin IHC Staining in Bladder Tissues**. The normal bladder tissue shown in **A**, **B**, **C**, and **D**, was part of a paraffin slide panel containing normal human tissues (Multi-Tissue VI) obtained from BioChain Institute, Inc. (Hayward, CA). The TCC and matching non-cancer tissues shown in **E**, **F**, **G**, and **H**, were part of a Bladder Carcinoma TMA (ARY-HH0087) obtained from Folio Biosciences (Columbus, OH). The ARY-HH0087 TMA contained 80 tissue cores, each at 1.5 mm in diameter; with 40 cancer tissues and 40 matching or independent non-cancerous tissues. The IHC staining with a prostasin-specific polyclonal antibody was carried out as described previously [11]. Prostasin-specific positive staining is shown by the intense brown color in **A**, **B**, **C**, **E**, and **H**. **D. Normal w/o Pro Ab**: IHC was performed with the pre-immune rabbit serum in place of the prostasin antiserum, serving as a negative control. Images were taken with various objective lenses (**O.L**.) as indicated in the figure and a SONY DXC-950 3CCD camera using a 0.45× coupler.

Using paraffin tissue sections of normal human epithelial tissues and tissue microarrays (TMA) of bladder cancer patients, we performed immunohistochemical (IHC) evaluation of prostasin expression. Prostasin is expressed specifically in the urothelial cells across all layers of the transitional epithelium in the normal tissues, shown as the intense brown-colored staining in Figures 1A, B, and 1C. No staining was present when the pre-immune serum was used in place of the human prostasin anti-serum (Figure 1D). In normal, or matched non-cancerous tissues on the TMA, prostasin expression was positive in 91.7% (33/36) of the cases. In low-grade (Grade I and II) transitional cell carcinomas, prostasin expression was significantly down-regulated, positively stained in 62.5% (5/8) and 35.3% (6/17), of the cases evaluated, respectively. In high-grade (Grade III) transitional cell carcinomas, only 16.7% (2/12) of the cases were positive for prostasin staining. In Figure 1 we show representative staining with prostasin expression in Grade-I TCC (**E**), absence of expression in Grade-II (**F**) and Grade-III TCC (**G**), and expression in matched non-cancerous urothelium (**H**, matching tissue from patient in **G**). The results of the TMA prostasin IHC staining are summarized in Table 1. The average prostasin staining scores among the tumor grade groups and the non-cancerous group were statistically different from one another between any two groups.

**Table 1:** Summary of TMA Prostasin IHC Staining Results.

Prostasin IHC Staining in Bladder TMA	Grade I	Grade II	Grade III	Normal or Matching Non-Cancerous
Number of Cases	8	17	12	36

Positive Cases	5	6	2	33

Percent Positive	62.5	35.3	16.7	91.7

The ARY-HH0087 Bladder Carcinoma TMA contained 40 cases of cancer, of which, 37 were TCC of various grades. Only the data on the 37 cases of TCC are included in this table. The TMA also contained 40 non-cancerous tissues, of which, 36 presented quality urothelial morphology for evaluation. Prostasin-specific staining was noted as positive (with a score of 1.0, as shown in Figure 1E or 1H) or negative (with a score of 0, as shown in Figure 1F, or 1G), as described in **Methods**. The IHC scores of the different tumor grade groups and the non-cancerous group were evaluated by ANOVA (single-factor) with ranked prostasin staining data (rank 1 = no staining, rank 2 = positive staining), yielding an F ratio of 15.46 (F critical = 2.74, p = 8.57E-08, and within-group degrees of freedom for error at 69). The average prostasin staining scores between any two groups were statistically different as determined by the Tukey-Kramer method.

### Prostasin protein is expressed in the normal urothelial cell line UROtsa, and in TCC cell lines with epithelial morphology but not mesenchymal morphology

We further evaluated prostasin protein expression in a panel of urothelial cell lines, including a normal immortalized urothelial cell line UROtsa, and TCC cell lines HT-1376, J82, RT4, T24, KU-7, 253J P, 253J B-V, UM-UC-3, -5, -6, -9, -10, -12, -13, and -14.

**Figure 2:**
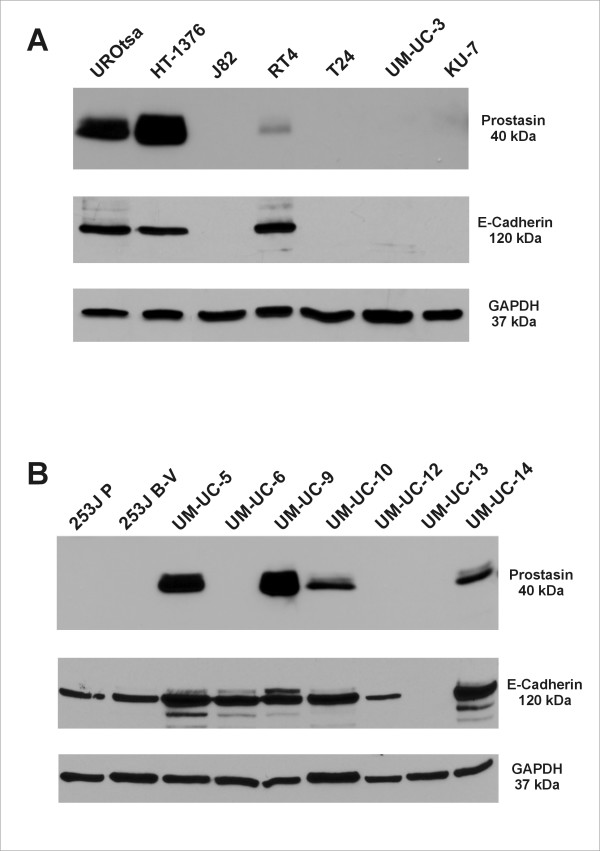
**Prostasin Expression in Urothelial Cell Lines**. Western blots were performed for protein expression evaluation of prostasin, E-cadherin, and GAPDH (as a control for sample loading) in the urothelial cell lines. Sample loading order was as indicated in the figure, 20 μg of total protein of each cell lysate were loaded in each lane. The nitrocellulose membrane was blotted or re-blotted separately for each target protein.

**Figure 3:**
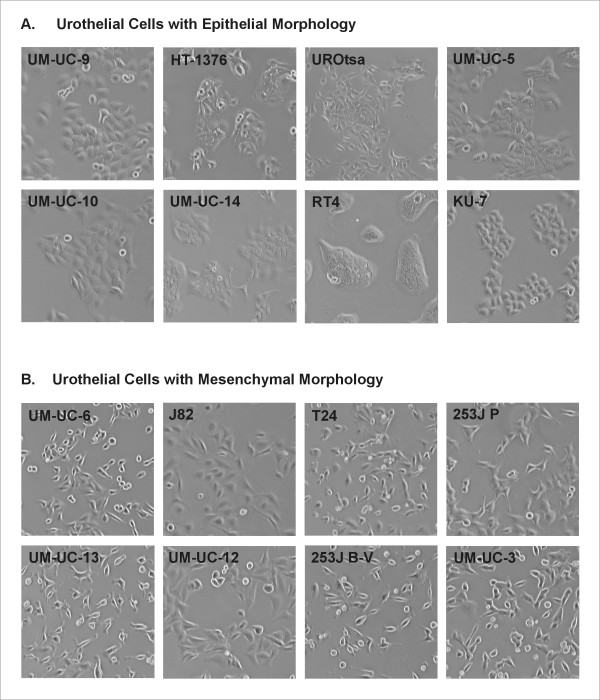
**Urothelial Cell Morphology**. Sub-confluent cultures of the urothelial cells were used for phase-contrast photography. **A**. Urothelial cells with epithelial morphology are positive for prostasin expression, except for KU-7 (see Figure 4). **B**. Urothelial cells with mesenchymal morphology are all negative for prostasin expression (see Figure 4).

Prostasin protein expression was evaluated by western blot analysis in the normal human urothelial cell line UROtsa, and a panel of TCC cell lines from the ATCC, HT-1376, J82, RT4, T24, and UM-UC-3, as well as the TCC cell line KU-7. As shown in Figure 2A, the UROtsa cells and the TCC cell line HT-1376 express an abundance of the prostasin protein, while the TCC cell line RT4 expresses a low but appreciable amount. The J82, T24, UM-UC-3, and KU-7 TCC cell lines were negative for prostasin protein expression by western blot analysis. The prostasin-positive UROtsa, HT-1376, and RT4 cells appear epithelial-like in 2-D cultures on plastic dishes, as islands of flat and polygonal-shaped cells with well defined cell-cell contacts in low-density cultures, as shown in Figure 3. These three cell lines all express an abundant level of the E-cadherin protein (Figure 2A). The prostasin-negative J82, T24, and UM-UC-3 cells all appear as spindle-shaped single cells or clusters without well defined cell-cell contacts in low-density cultures (Figure 3), i.e., fibroblastic or mesenchymal, and do not express E-cadherin at the protein level (Figure 2A). The only exception is the prostasin-negative KU-7 cell line, which does not express E-cadherin but is epithelial in appearance (Figure 2A and Figure 3), as previously noted by Black et al. [21].

We also evaluated prostasin protein expression in a panel of TCC cell lines from the MD Anderson Cancer Center (MDA) (Houston, TX). In Figure 2B, we show that the UM-UC-5, -9, -10, and -14 TCC cell lines express very high (UM-UC-5, -9) or intermediate levels (UM-UC-10, -14) of the prostasin protein. The 253J P, 253J B-V, UM-UC-6, -12, and -13 TCC cell lines do not express the prostasin protein. The E-cadherin protein expression information on the MDA TCC cell lines was recently reported by Black et al. [21]. The prostasin-positive MDA TCC cell lines, UM-UC-5, -9, -10, and -14, were shown to express a high abundance of E-cadherin at the protein level, and were also all epithelial in appearance in culture [21]. We have confirmed the observations on E-cadherin protein expression and morphology for these four cell lines (Figure 2B and Figure 3). Two of the prostasin-negative MDA TCC cell lines, UM-UC-6 and -13, were shown not to express E-cadherin and to have mesenchymal-like morphology in culture by Black et al. [21]. These two cell lines also appeared mesenchymal in culture in our hands (Figure 3), but the UM-UC-6 cells expressed E-cadherin at the protein level (Figure 2B). The prostasin-negative 253J P, 253J B-V and UM-UC-12 cell lines express the E-cadherin protein at low to medium levels (Figure 2B, and [21]) and are mesenchymal-like in culture (Figure 3).

**Figure 4:**
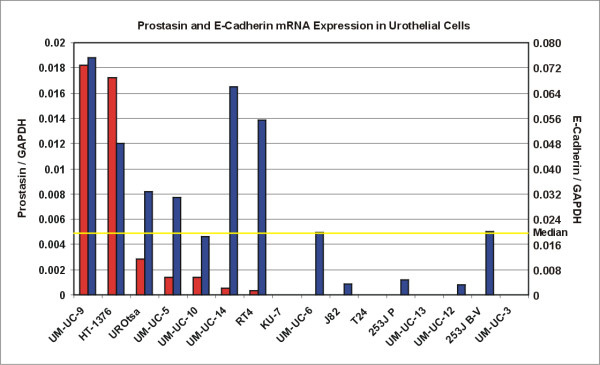
**Prostasin and E-cadherin mRNA Expression in Urothelial Cells Evaluated by qRT-PCR**. The red and blue data columns represent prostasin and E-cadherin mRNA expression, respectively, shown as relative levels per GAPDH mRNA copy. The data columns were sorted by the relative prostasin mRNA levels in the descending order from left to right. The yellow horizontal line indicates the median E-cadherin mRNA level.

For comparisons of gene expression at the transcriptional level (mRNA), we performed reverse-transcription and real-time quantitative PCR (qRT-PCR) analysis of the urothelial cell lines for prostasin, E-cadherin, and EGFR expression. The results for prostasin and E-cadherin are shown in Figure 4. The data columns were sorted by the relative prostasin mRNA levels (per GAPDH mRNA copy) in the descending order. While the qRT-PCR analysis yielded a copy number readout for every cell line, an arbitrary "zero" cut-off for prostasin mRNA expression in the data presentation was set with the KU-7 cell line, which does not express detectable levels of the prostasin protein (Figure 2A). Among the seven cell lines positive for prostasin mRNA, all but one (UM-UC-10) expressed the E-cadherin mRNA at above the median level (Figure 4). Among the nine cell lines negative for prostasin mRNA, all but two (UM-UC-6 and 253J B-V) expressed the E-cadherin mRNA at below the median level, and four lines were negative (Figure 4). The data on urothelial and TCC cell line prostasin and E-cadherin expression, and cell morphology are summarized in Table 2. The relative EGFR mRNA expression levels were compared among the urothelial and TCC cell lines and the results are listed in Table 2, as well.

**Table 2:** Prostasin Association with the Epithelial Phenotype in Urothelial Cells.

Urothelial Cell Lines	Prostasin Protein Levels	Prostasin mRNA Levels	E-Cadherin Protein Levels	E-Cadherin mRNA Levels	Morphology (2-D, Plastic)	Prostasin -96 CpG Methylation	EGFR mRNA Levels
UM-UC-9	+++++	+++++++++	+++	+++++++++	Epithelial	+/-	+++++++

HT-1376	+++++	+++++++++	+++	++++++	Epithelial	-	50× +

UROtsa	+++	+++	+++	++++	Epithelial	-	> 20× +

UM-UC-5	+++	++	+++	++++	Epithelial	-	> 250× +

UM-UC-10	++	++	+++	++	Epithelial	-	++++

UM-UC-14	++	+	+++	++++++++	Epithelial	-	+++

RT4	+	+	+++	+++++++	Epithelial	-	+++++

KU-7	-	-	-	-	Epithelial	+	++++

UM-UC-6	-	-	+++	++	Mesenchymal	+	> 10× +

J82	-	-	-	+	Mesenchymal	+	+++++++++

T24	-	-	-	-	Mesenchymal	+	++

253 J P	-	-	++	+	Mesenchymal	+	++++

UM-UC-13	-	-	-	-	Mesenchymal	+	++++

UM-UC-12	-	-	+	+	Mesenchymal	+	+++++++

253J B-V	-	-	++	++	Mesenchymal	+	> 10× +

UM-UC-3	-	-	-	-	Mesenchymal	+	+++

### The prostasin gene promoter is unmethylated in prostasin-expressing urothelial or TCC cell lines, but hypermethylated in TCC cell lines with down-regulated or lack of prostasin expression

**Figure 5:**
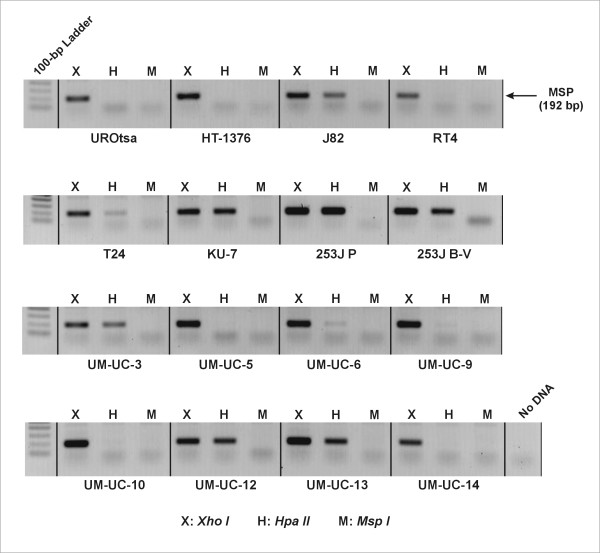
**Prostasin Promoter DNA Methylation in Urothelial Cell Lines**. MSP was performed to determine the methylation state at the -96 CpG dinucleotide in the prostasin gene promoter region. The cell types are indicated under the sectioned images of the MSP results. The restriction enzymes used for digestion of the genomic DNA prior to MSP are indicated above the sectioned images for each cell type; X: *Xho I*, H: *Hpa II *(methylation-sensitive), M: *Msp I *(methylation-insensitive).

We have previously shown that prostasin down-regulation in invasive human prostate and breast cancer cell lines was partially caused by promoter DNA hypermethylation [15,16]. In the CpG-rich promoter region of the prostasin gene, methylation state at the CpG dinucleotides at position -96, part of the restriction endonuclease recognition/cutting sequence for Hpa II/Msp I, was directly correlated to prostasin expression state [15,16]. We used a methylation-specific PCR (MSP) method to evaluate the methylation states of the -96 CpG in the urothelial cell lines. The MSP amplicon is 192 bp in length, and is expected from the Xho I-digested DNA as this enzyme does not cut in the amplicon region. Amplification of the MSP amplicon from the Hpa II-digested DNA occurs only when the DNA is methylated at the -96 CpG as Hpa II is methylation sensitive and will only digest unmethylated but not methylated DNA. No amplification of the MSP band is expected from the Msp I-digested DNA as this enzyme is insensitive to DNA methylation state at the -96 CpG and will cut either unmethylated or methylated DNA. As shown in Figure 5, the prostasin-expressing cell lines, UROtsa, HT-1376, RT4, UM-UC-5, -10, and -14 are not methylated at this site, as the MSP amplicon is absent from the Hpa II-digested DNA of these cell lines under the specific experimental conditions. The cell lines negative for prostasin protein expression, J82, T24, KU-7, 253J P, 253J B-V, UM-UC-3, -6, -12, and -13 are methylated to various degrees at the -96 CpG, as indicated by the MSP amplicon signals from the Hpa II-digested DNA of these cell lines. The UM-UC-9 cell line expresses a high abundance of prostasin mRNA and protein, but a very weak MSP amplicon signal was detected from the Hpa II-digested DNA of this cell line.

**Figure 6:**
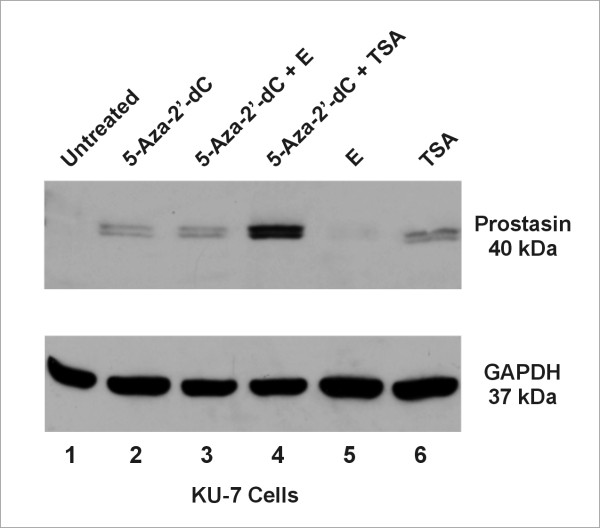
**Demethylation or HDAC Inhibition Reactivates Prostasin Expression in TCC Cells**. KU-7 cells were treated with **5-Aza-2'-dC**, Trichostatin A (**TSA**) or equal volumes of 95% ethanol (**E**) (solvent control for TSA), or in combinations as indicated in the figure, before western blot analysis for prostasin protein expression. The results are representative of multiple repeat experiments.

To determine if promoter DNA hypermethylation is a potential mechanism for prostasin expression silencing in TCC, we treated the KU-7 cells with the demethylation agent 5-aza-2'-deoxycytidine (5-Aza-2'-dC). We chose to use the KU-7 for this experiment because it is the only cell line with an epithelial morphology among all prostasin-negative TCC cell lines, and it has the highest prostasin mRNA copy number among the prostasin-negative lines (Figure 4). Demethylation treatment alone resulted in reactivation of prostasin protein expression (Figure 6, **Lane 2, versus Lane 1**). The histone deacetylase (HDAC) inhibitor trichostatin A (TSA) was also able to reactivate prostasin protein expression independently of demethylation (Figure 6, **Lane 6**). There was a synergistic effect on the reactivation of prostasin protein expression when HDAC inhibition was combined with demethylation (Figure 6, **Lane 4**). Treating the cells with ethanol (E) alone did not reactivate prostasin expression (Figure 6, **Lane 5**), nor had any synergistic effects on 5-Aza-2'-dC (Figure 6, **Lane 3**).

### Re-expression of prostasin or a serine active-site mutant variant in KU-7 was associated with E-Cadherin mRNA up-regulation

**Figure 7:**
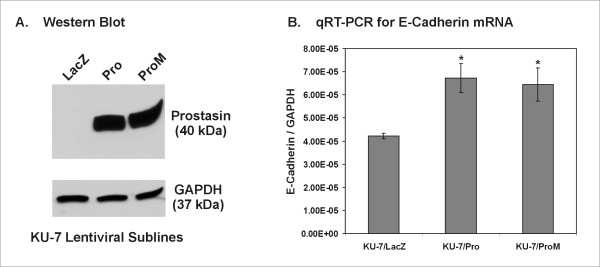
**Re-expression of Prostasin and Protease-inactive Variant in KU-7 Cells Up-regulates E-cadherin Transcription**. **A**. Western blots for prostasin and GAPDH for the KU-7 lentiviral sublines. Sample loading order was as indicated in the figure, 20 μg of total protein from each cell lysate were loaded in each lane. The nitrocellulose membrane was re-blotted separately for each target protein. **B**. qRT-PCR analysis of E-cadherin mRNA expression in the KU-7 lentiviral sublines. The data columns represent E-cadherin mRNA expression, shown as relative levels per GAPDH mRNA copy. The asterisks indicate a statistical difference (p < 0.05, n = 3) in the expression levels when compared with that of the KU-7/LacZ subline.

To determine if prostasin re-expression in a TCC cell line could result in E-cadherin up-regulation, as we have observed previously for the human prostate cancer cell line PC-3 [8], we infected the KU-7 cell line with lentiviruses driving the expression of the wild-type human prostasin (Pro), or a serine active-site mutant variant prostasin (ProM). As shown in Figure 7, when the wild-type or the mutant prostasin protein was expressed at similar levels (Figure 7A), the E-cadherin mRNA was up-regulated by ~50% by the wild-type or the mutant prostasin (Figure 7B).

## Discussion

We have shown that the prostasin serine protease is abundantly expressed in the normal terminally differentiated human urothelium, but significantly down-regulated in high-grade TCC (Figure 1 and Table 1). This is not surprising given prostasin's required role in terminal epithelial differentiation [9]. This association of prostasin expression and epithelial differentiation also holds true in the urothelial and cancer cell lines that we have evaluated, as shown in the summary of data in Table 2. The following observations were made upon examinations of the data in the present study. The presence of prostasin protein expression (in 7 of 16 cell lines evaluated) is associated with the highest levels of prostasin mRNA expression and there is a direct correlation of the prostasin protein expression levels and the prostasin mRNA expression levels. Prostasin protein or mRNA expression in the urothelial cells is 100% associated with the epithelial morphology, and with the most abundant E-cadherin protein expression (+++). Prostasin expression is also strongly associated with above-median level E-cadherin mRNA expression, in 6 out of 7 lines (Figure 4). Conversely, a lack of prostasin protein or mRNA expression in 9 of the 16 cell lines is strongly associated with the mesenchymal morphology (8 out of 9), with only one exception (KU-7). A lack of prostasin protein or mRNA expression is also strongly associated with below-median level E-cadherin mRNA expression, in 7 out of 9 lines (Figure 4). Only one cell line with a high abundance of E-cadherin protein expression (+++), UM-UC-6, is negative for prostasin expression. We did not, however, find a good correlation between prostasin expression and EGFR mRNA expression in the urothelial cell lines, except to note that the highest levels of EGFR mRNA expression were observed among the prostasin-positive cell lines, for example, UROtsa, HT-1376, and UM-UC-5. Black et al. [21] reported recently that resistance to the anti-EGFR monoclonal antibody drug cetuximab displayed by the TCC cell lines correlated with their E-cadherin protein expression state and cell morphology. With a strong correlation to the epithelial morphology and to E-cadherin expression, prostasin expression may also correlate with urothelial cancer sensitivity to cetuximab. This potential correlation will be investigated in future studies.

Prostasin promoter DNA hypermethylation is an epigenetic mechanism of prostasin expression silencing in human gastric, breast, and prostate cancer cells [14-16]. In this study, we showed that methylation of the -96 CpG dinucleotide in the promoter region is associated with prostasin expression silencing in the TCC cell lines. By the ratio of the MSP amplicon signal from the Hpa II-digested DNA to the signal from the Xho I-digested DNA (Figure 5), we could see that the prostasin promoter -96 CpG dinucleotide is methylated to various degrees in the TCC cell lines negative for prostasin expression. In most cases, the ratio suggests a heterogeneous state of methylation, i.e., the amplicon signal is stronger from the Xho I-digested DNA than that from the Hpa II-digested DNA. In the 253J P cells, the amplicon signal from the Hpa II-digested DNA is as strong as that from the Xho I-digested DNA, suggesting a homogeneous methylation state at the -96 CpG. On the other hand, the UROtsa and all TCC cell lines expressing the prostasin mRNA and protein, except for one (UM-UC-9), did not present -96 CpG methylation at the sensitivity level of our MSP assay. The UM-UC-9 cell line with abundant prostasin mRNA and protein expression had a weak but detectable signal of the MSP amplicon from the Hpa II-digested DNA. This is consistent with our previous findings that heterogeneous methylation at this site is permissive for prostasin protein expression, as was seen in the MDA-MB-453 human breast cancer cell line [15]. Clearly promoter region CpG dinucleotide methylation is not the only mechanism by which prostasin expression is regulated in epithelial or cancer cells. Within our MSP assay's sensitivity, the UM-UC-6 cell line is only marginally more methylated than the UM-UC-9 at the -96 CpG (Figure 5), yet the UM-UC-6 is negative for prostasin mRNA or protein expression while the UM-UC-9 expresses an abundance of prostasin mRNA and protein (Figure 2B and Figure 4). Other factors that could also regulate prostasin expression in the urothelial or urothelial cancer cells, such as the sterol regulatory element-binding proteins (SREBP's) and the SNAIL family transcription repressors [23], will be investigated in future studies. In the KU-7 cells, demethylation with 5-Aza-2'-dC or HDAC inhibition with TSA independently restored prostasin protein expression to a level detectable by western blot analysis, and to a greater level when the two agents were combined (Figure 6). This synergistic effect of 5-Aza-2'-dC and TSA on prostasin expression was also observed for the prostasin/E-cadherin double-negative T24 cell line, which is mesenchymal in morphology, with an up-regulation of the prostasin mRNA (data not shown). The E-cadherin mRNA in the T24 cells was also up-regulated by 5-Aza-2'-dC or TSA, and more robustly up-regulated by their combination (data not shown). We could not, however, attribute the E-cadherin mRNA up-regulation solely to the up-regulation of prostasin because these two epigenetic modulating agents have a direct impact on the E-cadherin promoter [24,25]. DNA demethylation agents and HDAC inhibitors, though, could be used as intravesical drugs for restoring prostasin expression in TCC and suppressing tumor invasion and metastasis once an anti-invasion role for prostasin in TCC is established.

We had previously shown with the PC-3 human prostate cancer cell line, that re-expression of the wild-type prostasin or a serine protease-inactive mutant could up-regulate E-cadherin expression via a transcriptional mechanism [8]. This transcriptional up-regulation of E-cadherin by prostasin or the protease-inactive variant could be recapitulated in the KU-7 cells (Figure 7), though not as robust as that seen previously with the PC-3 cells [8]. The extent by which E-cadherin transcription is up-regulated by prostasin re-expression could be impacted by epigenetic modifications in the E-cadherin promoter, events that are common in cancer cells [24,25]. We also performed prostasin expression silencing by using a prostasin-specific siRNA previously shown to effectively knock-down its expression [26]. We knocked-down prostasin expression by at least 50% and up to 75%, in five urothelial cell lines, UROtsa, HT-1376, RT4, UM-UC-5, and UM-UC-9, but the prostasin expression knock-down was not associated with E-cadherin down-regulation (data not shown). We have observed in the TCC cell lines, a low level of prostasin expression could still be associated with a high abundance of E-cadherin expression, as seen with the RT4 cells (Figure 2B and Figure 4). The prostasin expression knock-down may not have been sufficient to affect E-cadherin expression. During urothelial tumorigenesis, prostasin expression down-regulation is likely a progressive event. This hypothesis is supported by the IHC data, showing a progressive loss of prostasin expression from non-cancerous tissues to TCC of increasing grades (Table 1). The association between prostasin expression state and cell morphology is manifested at the two end points of this progression, i.e., the starting end of epithelial morphology and prostasin/E-cadherin expression, and the EMT end of mesenchymal morphology and loss of prostasin with reduced or loss of E-cadherin expression. It may require a complete loss of prostasin expression over many generations of cell division to have a significant impact on E-cadherin expression and cell morphology seen in the prostasin-negative TCC cell lines.

## Conclusion

Expression of prostasin in the urothelial cells is associated with the epithelial state, marked by abundant E-cadherin expression. Absence of prostasin is associated with the epithelial-mesenchymal transition (EMT), marked by a loss of or a reduced E-cadherin expression. EMT and EMT-associated molecular changes are underlying mechanisms of malignant progression of TCC, such as gain of invasive potential and resistance to anti-EGFR therapy. Future research is warranted to address if prostasin may be used as a therapeutic agent for treating invasive and chemo-resistant TCC cells that have undergone an epithelial-mesenchymal transition.

## Competing interests

The authors declare that they have no competing interests.

## Authors' contributions

LC carried out all of the IHC work and the western blot analysis. NJV performed the lentiviral infection and analysis of the KU-7 cells re-expressing prostasin. KXC performed the methylation-specific PCR, prostasin expression reactivation, and the qRT-PCR experiments. LC and KXC contributed equally in the conception and writing of the paper, and all authors approved the final manuscript.

## Pre-publication history

The pre-publication history for this paper can be accessed here:

http://www.biomedcentral.com/1471-2407/9/377/prepub
